# Fibrous hamartoma of infancy: a case report

**DOI:** 10.3389/fped.2026.1948547

**Published:** 2026-09-25

**Authors:** Ninoslav Begovic, Milos Vasic, Milica Malis, David Pavicevic, Gordana Samardzija, Lidija Andrejevic, Ivan Dizdarević, Natasa Vlahovic, Marko Jevric, Borislav Toskovic

**Affiliations:** 1 Department of Pediatric Orthopedics and Traumatology, Institute for Health Care of Mother and Child of Serbia, “Dr Vukan Cupic”, Belgrade, Serbia; 2 Faculty of Medicine, University of Belgrade, Belgrade, Serbia; 3 Department of Plastic and Reconstructive Surgery, Institute for Health Care of Mother and Child of Serbia, “Dr Vukan Cupic”, Belgrade, Serbia; 4 Department of Pathology, Institute for Health Care of Mother and Child of Serbia, “Dr Vukan Cupic”, Belgrade, Serbia; 5 Department of Cardiothoracic Surgery, Institute for Health Care of Mother and Child of Serbia, “Dr Vukan Cupic”, Belgrade, Serbia; 6 Department of Physical Therapy and Rehabilitation, Institute for Health Care of Mother and Child of Serbia, “Dr Vukan Cupic”, Belgrade, Serbia; 7 Clinic of Surgical Oncology, Institute of Oncology and Radiology of Serbia, Belgrade, Serbia; 8 Clinical Hospital “Bezanijska Kosa”, Belgrade, Serbia

**Keywords:** differential diagnosis, fibrous hamartoma of infancy, MRI diagnostic, pediatric surgery, soft tissue mass

## Abstract

**Introduction:**

Fibrous hamartoma of infancy is a rare benign mesenchymal soft-tissue tumor characterized by a triphasic organoid architecture. Because its clinical and imaging features are variable, fibrous hamartoma of infancy may mimic inflammatory, vascular, benign fibro-fatty, intermediate, or malignant soft-tissue lesions.

**Case description:**

We report an approximately 6-month-old boy with a painless, progressively enlarging subcutaneous mass in the right axillary/medial upper-arm region. The lesion was initially interpreted as BCG-associated lymphadenitis; aspiration yielded no purulent material, and serial ultrasonography demonstrated progressive enlargement. Magnetic resonance imaging revealed a 67  ×  40 × 58 mm well-circumscribed subcutaneous mass with marked contrast enhancement and no definite diffusion restriction or muscle invasion; infantile myofibroma and an intermediate soft-tissue lesion were considered. Complete gross excision was achieved while preserving the visualized muscle and neurovascular structures. Histopathologic examination demonstrated the characteristic triphasic pattern of fibrous hamartoma of infancy, and the immunohistochemical findings supported the diagnosis.

**Discussion:**

This case illustrates how sequential clinical assessment, multimodal imaging, operative findings, and histopathologic correlation can resolve a broad differential diagnosis in an infant with a rapidly enlarging soft-tissue mass.

**Conclusion:**

Fibrous hamartoma of infancy should be considered in infants with a painless, enlarging axillary or upper-extremity mass, particularly when imaging findings are nonspecific. Histopathologic confirmation is essential to establish the diagnosis and avoid unnecessarily aggressive oncologic treatment.

## Introduction

Fibrous hamartoma of infancy (FHI) is an uncommon benign fibroblastic/myofibroblastic tumor of the superficial soft tissues that usually arises in the subcutis or lower dermis. Classic literature demonstrates a strong predilection for early infancy: in a review of 197 reported cases, 91% occurred during the first year of life and 23% were congenital, with a male predominance and frequent involvement of the axilla, upper arm, upper trunk, inguinal region, and external genitalia (1). Subsequent large clinicopathologic series confirmed the typical age and anatomic distribution while demonstrating considerable morphologic variability (2, 3). More recent clinicopathologic and molecular studies have further supported FHI as a distinctive benign mesenchymal neoplasm with a characteristic triphasic architecture (4).

Preoperative diagnosis may be challenging because the clinical findings are nonspecific and the relative proportions of adipose, fibroblastic/myofibroblastic, and primitive myxoid components vary substantially among lesions. Ultrasonography (US) is usually the first-line imaging modality for a superficial soft-tissue mass in a child. Radiologic studies have described heterogeneous subcutaneous lesions with alternating echogenic and hypoechoic components, sometimes forming serpentine, semicircular, or fence-like patterns (5–7). Magnetic resonance imaging (MRI) can further delineate lesion extent and tissue composition when ultrasonographic findings are indeterminate, although MRI appearances are variable and may still be insufficient to establish the diagnosis (5, 7).

Accordingly, the differential diagnosis in an infant may include BCG-associated regional lymphadenitis or other inflammatory lymphadenopathy, infantile hemangioma or vascular malformation, infantile myofibroma, lipoblastoma/lipoblastomatosis, lipofibromatosis, desmoid-type fibromatosis, giant cell fibroblastoma/dermatofibrosarcoma protuberans spectrum, infantile fibrosarcoma, and rhabdomyosarcoma (2, 8–12). Distinguishing among these entities is clinically important because management may range from observation or targeted treatment to local excision or oncologic therapy.

Complete gross local excision remains the mainstay of treatment. However, contemporary data suggest that microscopically positive margins are relatively common and do not necessarily mandate aggressive re-excision, as clinically significant recurrence appears to be uncommon (13). We report an infant with an enlarging axillary/upper-arm FHI that initially mimicked BCG-associated lymphadenitis and subsequently raised vascular and neoplastic diagnostic considerations.

## Case presentation

A previously healthy male infant, born at term after an uncomplicated pregnancy and vaginal delivery, first presented to our plastic surgery clinic at approximately 6 months of age after his parents noticed swelling in the right axillary region that morning. He was afebrile, feeding and growing normally, and had no recent history of illness. BCG vaccination had been administered approximately one month earlier. Physical examination revealed a painless, mobile, non-fluctuant soft-tissue mass approximately 3 cm in diameter in the proximal medial aspect of the right upper arm, with normal overlying skin.

Initial ultrasonography demonstrated a well-defined 36 × 18 mm lesion immediately below the right axilla, with structural characteristics, echogenicity, and vascularity interpreted as consistent with an altered lymph node. Smaller reactive axillary lymph nodes were also present, without liquefaction. Given the recent BCG vaccination and axillary location, BCG-associated lymphadenitis was initially suspected. Needle aspiration yielded no purulent material. A 7-day course of amoxicillin/clavulanate was prescribed, and clinical and ultrasonographic follow-up was arranged.

Two weeks after the initial presentation, ultrasonography demonstrated enlargement of the lesion to 44 × 21 × 39 mm. The lesion was heterogeneous and predominantly hypoechoic, with hyperechoic band-like components containing color Doppler signals, and remained without liquefaction. Because the mass persisted, surgical excision was considered. At a subsequent review approximately two weeks later, the lesion remained present, and excision with histopathologic examination was planned. Over the following four weeks, the lesion enlarged by a further approximately 20%, prompting MRI before surgery (Figure 1A).

**Figure 1:**
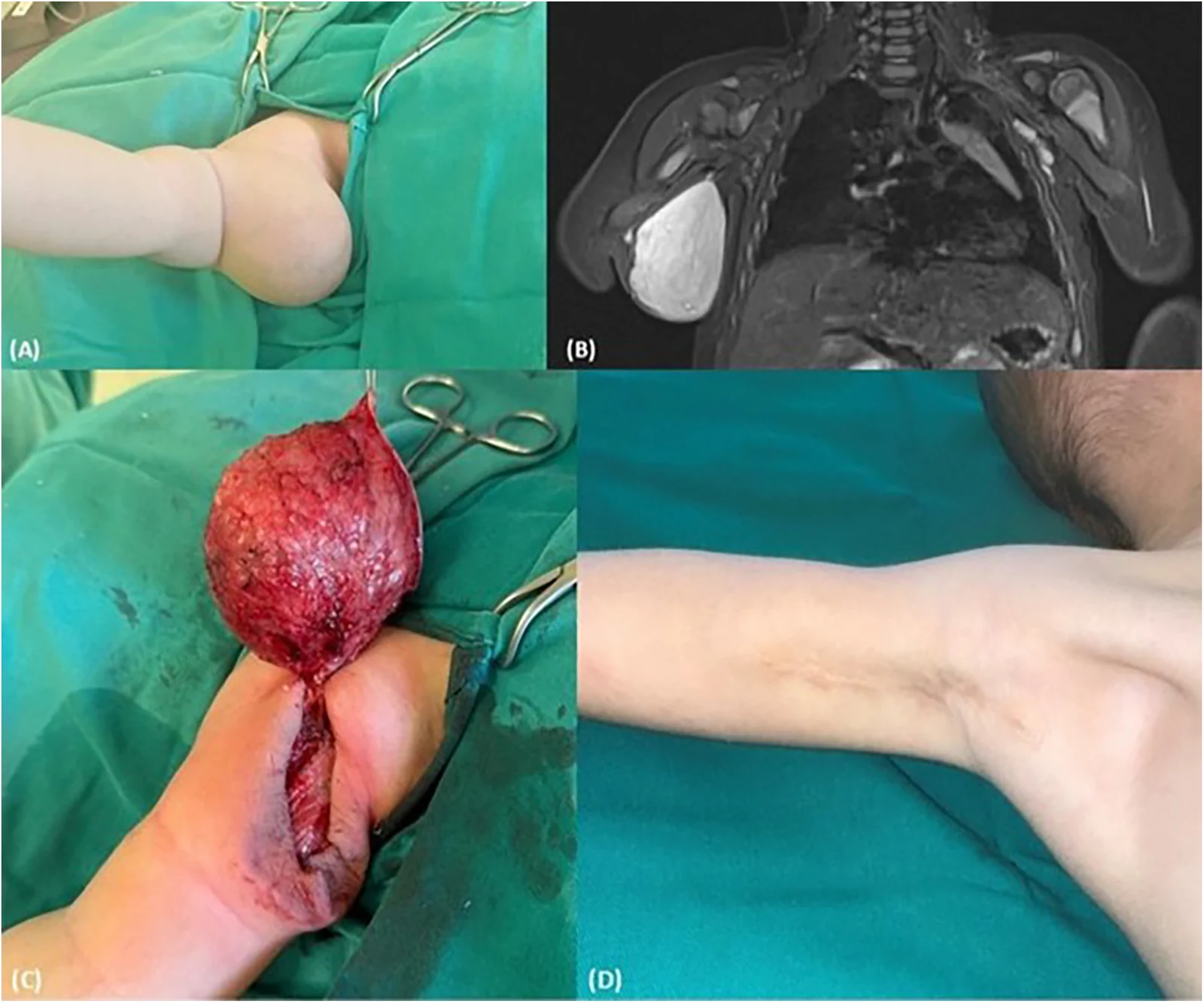
Clinical course and surgical management of the lesion. **(A)** Preoperative clinical appearance of the painless, well-circumscribed subcutaneous mass in the proximal medial aspect of the right upper arm, immediately below the axilla. **(B)** Magnetic resonance image of the right axillary/upper-arm mass, demonstrating a sharply demarcated subcutaneous lesion extending from the axilla along the medial upper arm, with marked post-contrast enhancement and no definite invasion of the adjacent muscle. **(C)** Intraoperative appearance of the lesion. The basal portion of the mass was adherent to the deeper soft tissues. Careful dissection was continued to the level of the muscle and neurovascular structures, which were clearly identified and preserved, allowing complete gross excision. **(D)** Clinical appearance approximately 10 months after surgery. The postoperative scar is flat, level with the surrounding skin, and normally pigmented, with no clinically evident recurrent swelling. On examination, the child had a full, pain-free range of motion of the right upper extremity.

Approximately 10 weeks after the initial presentation, MRI demonstrated a 67 × 40 × 58 mm oval, sharply demarcated, encapsulated mass within the subcutaneous fat immediately below the right axilla and extending along the medial upper arm (Figure 1B). The lesion was isointense to adjacent muscle on T1-weighted images, mildly hyperintense to muscle on T2-weighted images, hyperintense on STIR images, and showed relatively homogeneous, marked post-contrast enhancement. The apparent diffusion coefficient was intermediate (1.26 ± 0.04 × 10–3 mm^2^/s), without clear diffusion restriction. No internal liquefaction or definite invasion of the adjacent muscle was identified. Reactive bilateral axillary lymph nodes were visualized separately from the mass. The radiologic differential diagnosis included infantile myofibroma and an intermediate soft-tissue lesion, and tissue diagnosis was recommended.

Approximately three months after the initial presentation, the child was admitted for planned evaluation and treatment. He remained afebrile and in good general condition. Hematologic consultation documented a well-circumscribed, elastic, painless axillary mass, with preserved shoulder and elbow motion and a prominent superficial vascular pattern overlying the lesion. Routine laboratory evaluation showed no evidence of an inflammatory response (CRP, 0.8 mg/L).

During the same hospitalization, the lesion was excised under general anesthesia. An ellipsoidal skin excision was made. Careful dissection exposed the basal portion of the lesion, which was adherent to the deeper soft tissues over a segment approximately 5 cm in length. Dissection was continued to the level of the muscle and neurovascular structures, which were clearly identified and preserved, allowing complete gross excision of the mass (Figure 1C). Excess skin was excised to facilitate wound reconstruction. Hemostasis was achieved, a drain was placed, and the wound was closed in layers. Blood loss was minimal, and the operative and anesthetic courses were uncomplicated. Macroscopically, the excised specimen was a 145-g tumor measuring 70 × 50 × 55 mm.

Histologically, the lesion was a relatively well-circumscribed subcutaneous tumor with an organoid architecture composed predominantly of intersecting fibroblastic spindle-cell areas within collagenized/hyalinized stroma, admixed with mature adipose tissue and rare myxoid nodules containing primitive spindle cells**.** No giant or floret-like cells, significant mitotic activity, necrosis, or calcification were identified. Immunohistochemically, the tumor cells were positive for vimentin; CD34 was positive in the myxoid nodules, with weak focal staining in the fibrous areas; SMA and calponin were positive in the fibrous and myxoid components; and caldesmon, desmin, ALK, cytokeratin, SOX10, beta-catenin, STAT6 and HHF-35 were negative. S-100 positivity was confined to the adipose tissue. Cyclin D1 and BCL2 showed nuclear staining in the majority of tumor cells, and the Ki-67 proliferation index was less than 5%. Taken together, the characteristic triphasic morphology and immunophenotype supported the diagnosis of FHI (2–4, 12) (Figures 2A–D).

**Figure 2:**
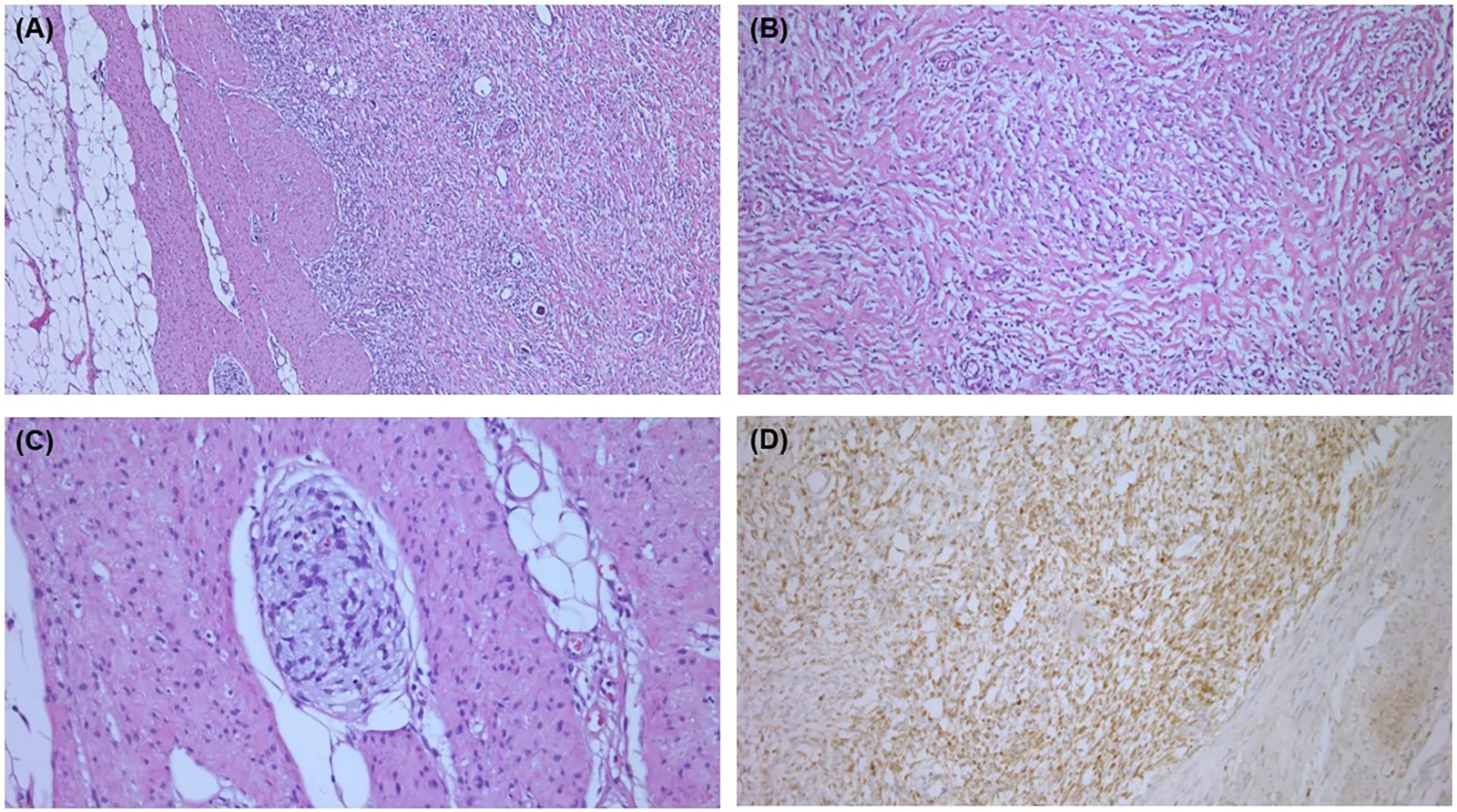
Histopathological finging of resected tumor. **(A)** A three-component tumor composed of fibrous tissue, mature adipose tissue and myxoid areas in an organoid pattern (Hematoxylin and Eosin, x50). **(B)** Intersection fascicles of fibrous tissue composed of fibroblastic cells within collagenized stroma predominate (Hematoxylin and Eosin, x100). **(C)** Myxoid nodule of spindle to stellate primitive cells (Hematoxylin and Eosin, x200). **(D)** Variable tumor expression of BCL2 (immunohistochemistry, x100).

The immediate postoperative course was uncomplicated. Approximately two weeks after surgery, the wound was dry and had healed by primary intention, with no signs of infection; the sutures were removed, and scar treatment was recommended. Definitive histopathologic confirmation was obtained approximately one month after surgery. At the most recent follow-up examination, approximately 10 months postoperatively, there was no clinical evidence of recurrent swelling (Figure 1D). The postoperative scar was flat, level with the surrounding skin, and normally pigmented. The child had a full, pain-free range of motion of the right upper extremity, with no functional impairment, and the parents reported no episodes of pain since the previous follow-up.

## Discussion

The present case illustrates how FHI, despite its benign biologic behavior, may enlarge substantially over a short period and mimic inflammatory, vascular, intermediate, or malignant soft-tissue disease (5, 7, 11). The patient's age, sex, and anatomic site were typical of FHI (1–4). Historical review data showed that 91% of reported cases occurred during the first year of life, while subsequent large series confirmed a male predominance and frequent axillary/upper-arm involvement (1–4). Recent reports continue to document FHI in very young infants, including a 2-month-old child and a 6-month-old infant with a pseudo angiomatous pattern (14, 15).

The initial consideration of an inflammatory process in our patient was clinically plausible. BCG-associated regional lymphadenitis is a recognized complication of BCG vaccination and may present as nonsuppurative lymphadenitis or progress to suppuration or abscess formation (8). In our patient, the recent vaccination, axillary location, and initial ultrasonographic interpretation as an altered lymph node supported this possibility. However, the absence of systemic illness, lack of liquefaction, absence of purulent material on aspiration, and progressive enlargement during observation argued against a simple BCG-related process. MRI subsequently demonstrated that the dominant lesion was a discrete subcutaneous mass, with reactive lymph nodes visualized separately.

A vascular lesion was also considered because Doppler signals were detected within hyperechoic bands on ultrasonography, a prominent superficial vascular pattern was subsequently documented clinically, and the mass showed marked contrast enhancement on MRI. Infantile hemangiomas and vascular malformations may show substantial vascularity, but their distinction relies on the combined clinical course and characteristic vascular imaging features (9, 10). In our case, histopathologic examination did not demonstrate a predominant vascular proliferation; instead, it revealed the characteristic triphasic pattern of FHI. Importantly, FHI itself may exhibit pseudoangiomatous morphology, which can represent an additional source of diagnostic confusion (3, 15, 16).

Retrospectively, the second ultrasonographic examination was particularly informative. The lesion was heterogeneous and predominantly hypoechoic, with hyperechoic band-like components. Published FHI series describe alternating echogenic and hypoechoic tissue reflecting the variable proportions of adipose tissue, fibrous tissue, and primitive mesenchyme and producing serpentine, semicircular, or fence-like patterns (5–7). Our examination was not originally interpreted as demonstrating a classic serpentine pattern, and this feature should therefore not be assigned retrospectively; nevertheless, the observed band-like heterogeneity overlaps with the imaging spectrum described for FHI (5–7). A 2026 Frontiers in Pediatrics series of 16 children is particularly relevant: all patients were initially misdiagnosed on ultrasonography, with lipoma and infantile hemangioma among the common incorrect diagnoses, underscoring the limitations of imaging alone (7).

MRI narrowed the differential diagnosis but did not establish a definitive diagnosis. The lesion was sharply circumscribed and showed no clear muscle invasion or diffusion restriction, which were reassuring features; however, marked enhancement and intermediate ADC values maintained concern for a neoplastic process. Infantile myofibroma was specifically proposed in the radiologic differential diagnosis. Histologically, however, infantile myofibroma characteristically shows a biphasic myofibroblastic pattern with a prominent perivascular/hemangiopericytoma-like component, whereas FHI displays an organoid combination of mature adipose tissue, fibroblastic fascicles, and primitive myxoid nodules, as observed in our tumor (2–4, 11).

Other benign and intermediate fibro-fatty lesions are important microscopic mimics of FHI (2, 11, 12). Lipoblastoma/lipoblastomatosis typically shows a lobulated immature adipocytic proliferation with lipoblasts, whereas lipofibromatosis is more infiltrative and lacks the complete triphasic pattern of FHI (2, 11). Desmoid-type fibromatosis is characterized by sweeping fibroblastic fascicles and commonly shows nuclear beta-catenin expression; beta-catenin was negative in our lesion (2, 11, 12). Giant cell fibroblastoma/DFSP-spectrum lesions may be CD34-positive; however, the absence of giant/floret-like cells and a monomorphic storiform proliferation, together with the characteristic triphasic architecture, favored FHI (2, 4, 11, 12). Inflammatory myofibroblastic tumor can be a microscopic mimic of FHI; however, the absence of a prominent inflammatory infiltrate and ALK negativity argued against this diagnosis. Cellular schwannoma may show overlapping spindle-cell morphology, but diffuse S-100 positivity was absent in our lesion. Solitary fibrous tumor may also enter the differential diagnosis because of its spindle-cell morphology and CD34 expression; however, STAT6 negativity argued against this diagnosis.

The most clinically consequential differential diagnoses are malignant tumors, particularly infantile fibrosarcoma and rhabdomyosarcoma (2, 11, 12). Because FHI itself may enlarge rapidly, rapid growth alone is not specific for sarcoma (2, 7, 11). In the present case, the absence of destructive infiltration on MRI, significant cytologic atypia, necrosis, and brisk mitotic activity, together with a low Ki-67 proliferation index and the organoid triphasic morphology, argued strongly against sarcoma. Desmin negativity provided further evidence against rhabdomyosarcoma (11, 12). Rare sarcomatous change has been reported in FHI; therefore, morphologically atypical cases warrant careful reassessment, and molecular testing may be useful when a competing diagnosis such as infantile fibrosarcoma remains a concern (2, 11, 17).

In this case, surgery was both diagnostic and therapeutic. The mass was adherent to the deeper soft tissues at its base, but the muscle and neurovascular structures were clearly identified and preserved during complete gross excision. This operative appearance is relevant because FHI may be locally infiltrative despite its benign biologic behavior (2, 13). Contemporary surgical data challenge the need for radical re-excision solely to achieve microscopically negative margins: in a 2024 series, 6 of 9 patients had positive margins, but only one developed local recurrence, suggesting that individualized surveillance may be reasonable after complete gross excision when further surgery would result in additional morbidity (13). Accordingly, complete gross local excision with preservation of function appears preferable to unnecessarily radical resection (2, 13).

The strengths of this report include serial clinical and ultrasonographic documentation of lesion growth, detailed MRI assessment, operative correlation, comprehensive histopathologic and immunohistochemical evaluation, and documented postoperative clinical follow-up. The principal limitations are the absence of a preoperative tissue diagnosis and the nonspecific imaging phenotype. The main educational message is that FHI should remain in the differential diagnosis of a rapidly enlarging axillary or upper-extremity mass in an infant, even when initial imaging suggests lymphadenopathy or a vascular or neoplastic lesion. Sequential reassessment and histopathologic correlation may help reduce diagnostic delay and avoid unnecessarily aggressive treatment (7, 13).

## Conclusion

FHI is an uncommon benign tumor of infancy with variable clinical and imaging appearances. In an infant with a painless, enlarging axillary or upper-arm mass, the differential diagnosis should include inflammatory lymphadenopathy, vascular lesions, benign and intermediate fibro-fatty tumors, and soft-tissue sarcomas. Ultrasonography and MRI are useful for defining lesion extent and guiding management but may not establish the diagnosis. Recognition of the characteristic triphasic histologic architecture is decisive. Complete gross local excision with preservation of adjacent functional structures is generally appropriate, whereas unnecessarily radical resection should be avoided in this biologically benign lesion.

## Patient perspective

The parents reported substantial anxiety after noticing the mass, particularly as it enlarged rapidly. Following surgery and confirmation of the benign diagnosis, they expressed relief that the lesion had been completely removed without apparent harm to their child. At the most recent follow-up, they reported that the child had experienced no pain since the previous follow-up visit and had no limitation in the use of the right upper extremity.

## Data Availability

The original contributions presented in the study are included in the article/Supplementary Material, further inquiries can be directed to the corresponding author.
